# Membership in team science institute enhances diversity of researchers’ collaboration networks

**DOI:** 10.1371/journal.pone.0322943

**Published:** 2025-05-23

**Authors:** William C. Barley, Ly Dinh, Lauren P. Johnson, Brian F. Allan

**Affiliations:** 1 Department of Communication, University of Illinois Urbana-Champaign, Urbana, IL, United States of America; 2 School of Information Science, University of South Florida, Tampa, FL, United States of America; 3 Department of Entomology, University of Illinois Urbana-Champaign, Urbana, IL, United States of America; Ingenio CSIC-UPV, SPAIN

## Abstract

Interdisciplinary scientific teams are subject to a complex constellation of potential benefits, such as enabling innovation, and challenges, such as increased conflict and failure. Given these tensions, scholars and practitioners are increasingly interested in the role that organizational policies and resources can play in potentially mitigating the challenges faced on interdisciplinary teams. We report results from quantitative case study of a research institute dedicated to providing resources to enable interdisciplinary scientific teams, to examine how joining an organization with resources devoted to interdisciplinarity affected researchers’ collaborations. We adopt bibliometric network techniques to explore the productivity and diversity of scientists’ collaborations before and after joining the institute. Generalized linear mixed-effect modeling shows a significant increase for researchers in their number of papers and co-authors after joining the institute. Comparison to a matched pair control group indicates researchers who joined the institute experienced a significantly greater increase in their diversity of co-authors, and no relative decrease in the number of papers produced, despite challenges inherent to interdisciplinary collaboration. These findings suggest institutional resources can operate to broaden collaboration diversity without harming researcher productivity, which has important implications for team science and science policy.

## Introduction

Harnessing institutional resources to support interdisciplinary collaborations in science is the subject of important recent discussions about science policy and practice. Science produced by teams plays an important role in creating impactful discoveries [1,2], especially when those teams are composed of individuals whose expertise represents diverse disciplinary perspectives [3–5]. In fact, bibliometrics research provides empirical evidence that interdisciplinary teams often produce higher-impact research, with interdisciplinarity measured through citation patterns, co-authorship networks, and disciplinary diversity in research outputs [6–8]. Conceptually, this effect is most frequently explained as the result of recombinant search processes allowing diverse teams to connect previously dissociated information to produce novel solutions [2,4,9]. Dedicated interdisciplinary research institutes have been established as one way to support such endeavors by investing significant resources to facilitate interdisciplinary collaborations. However, there is a critical need to understand whether and how the organizational structures and resources within these centers contribute to the success and productivity of interdisciplinary teams.

Mounting evidence suggests that dominant social forces in science inhibit interdisciplinary collaborations [10–14]. Interdisciplinarity presents unique challenges, such as extra communicative labor [10,11] and increased coordination costs [15]. Many institutions have cultures and incentives that may discourage interdisciplinary work. Multidisciplinary researchers can face harder scrutiny when being considered for promotion [12,13]. Junior scientists may face increasing difficulty in obtaining funding for their work, especially prior to achieving tenure [16]. This effect is compounded by funding agencies’ decreased propensity to fund interdisciplinary research [13]. Interdisciplinary researchers are also less productive, on average, than those focused on their home disciplines [10], as they require additional time to build trust, integrate diverse knowledge, and solve problems that span at least two disciplines [17]. Although important discoveries are increasingly interdisciplinary, they are also produced disproportionately by researchers who have access to resources and protections that allow cross-disciplinary exploration [14]. Thus, there is a growing need to understand the mechanisms by which interdisciplinary discovery occurs to better address the complex problems faced in contemporary society [18].

Given these challenges of interdisciplinary collaboration, scholars have explored how institutional resources can influence scientific productivity [11,19,20]. For example, institutional support through flexible funding and extended project timelines is crucial to enabling interdisciplinary work [21]. Administrative support, such as coaching to form meaningful teams, can increase likelihood of success [22–24]. Science studies scholars have also long recognized that infrastructural support, such as access to shared lab equipment and expert technical staff, can play an important role in facilitating diverse scientific collaboration [25–29].

However, the preponderance of empirical studies that examine the influence of research centers on interdisciplinarity examines individual research products, usually articles, as their focal object of analysis [20]. Focusing on independent paper outputs potentially masks that papers are produced by individuals embedded in teams and who produce a portfolio over time [5,30], and that the individuals who produce diverse outputs may do so at the expense of other aspects of their performance (e.g. research productivity). This study addresses this knowledge gap by focusing on researchers’ publications and the diversity of their co-authorship networks to capture the long-term impact of interdisciplinary center membership on individual career development, particularly in terms of collaboration patterns and research productivity over time.

Additionally, research on how joining interdisciplinary institutes affects individuals often lacks a control group comparison. Our analysis here addresses these concerns by using multiple diversity metrics and establishing a carefully selected control group for comparison to examine how joining an interdisciplinary science institute influenced the productivity and diversity of researchers’ collaboration, relative to those who remain in traditional disciplinary environments. This control group allows us to isolate the effects of interdisciplinary center membership, ensuring that observed differences are not simply due to natural career progression in academic research. Although our study draws upon a single-case design, the findings offer promising directions forward for scholars interested in the role of institutions in facilitating scientific teams.

## Materials and methods

The procedures for this study were evaluated by the Institutional Review Board of our university and received an exempt determination (IRB#21798). The IRB also approved a waiver of informed consent for this project. We took multiple measures to protect the individuals who are the subjects of this research, including de-identifying data during analysis, using a pseudonym to refer to the institute under study, and reporting our results in aggregate.

As a single-case design, it is important to describe the context in some detail. We adopted a pre-post comparison approach, with a control group, to examine researchers’ collaborative publication networks before and after joining the Nature Institute (NI; a pseudonym to protect researcher privacy), a large research institute in the biological sciences located at a large public university in the U.S.A., between 2004 and 2021. The NI supports its affiliates’ research via many forms of resources identified as important to fostering interdisciplinary research in prior literature, such as: seed funding to allow researchers to explore new potential interdisciplinary projects; access to cutting-edge measurement and analysis technologies with applications across multiple domains of the natural sciences; advice from technical staff who devote considerable time to building expertise in the application of the Institute’s technologies; pre- and post-award grant support from proposal writers who specialize in developing interdisciplinary funding applications; shared lab spaces intentionally designed to encourage cross-lab interactions; and an active seminar series exposing affiliates to cross-disciplinary research. Researchers affiliate with the NI via non-salary providing secondary appointments. All NI researchers maintain primary affiliations with their home disciplinary departments in addition to their relationship with the NI. NI policy requires a commitment to an interdisciplinary research agenda as a precondition to gaining a formal appointment with the NI.

These factors combine to make the NI an ideal site for examining multidisciplinary collaboration relationships. The Institute’s explicit commitment to interdisciplinary research and its enactment of multiple practices described in the literature on research institutes make it a useful case context for analysis. Further, the NI’s secondary appointment model allowed for the construction of a control-group comparison to researchers from the same home departments who would not go on to join the NI during their careers.

### Network construction

Our data consist of publication data from the careers of 394 researchers affiliated with the NI between 2004-2021. The analyses discussed here focus specifically on the subset of 208 individuals for whom we had a 4-year window of publications before and 4-year window of publications after the year they joined the NI, with the join year excluded from analysis. The join year was excluded to help account for any publication delay from work performed prior to joining the NI. This overall 9-year window was chosen based on a sensitivity analysis by which we determined that this window maximized the sample size and the duration of publications considered for each researcher before and after joining NI. Consistent with practices in bibliometrics research, where the choice of time window significantly influences a paper’s measured impact [31–33]), we conducted 12 experiments with varying time windows to determine the optimal range for our analysis. These experiments included testing pre- and post-join windows, as well as adjusting the total size of the gap surrounding the NI start date by excluding the join year and varying the gap length. Our time window of 4-years pre- and post-join is consistent with prior studies by Wang and colleagues [32], who found that longer windows (more than 1 year) better capture the citation impact of highly-cited papers. Abramo and colleagues’ [31] analysis of citation impact over an 8-year time window found that while time windows vary across disciplines, windows longer than 2 years provide a more reliable measure of research impact and productivity. The literature also suggests that it is important to have a gap between windows of measurement to account for publication delays [33,34], as the time lag between submission and publication has steadily increased across various disciplines. These studies demonstrate that publication delays tend to be shorter within the natural and physical sciences, such as those predominantly represented in our sample [33]. Hence, we concluded that at a gap length of one year would sufficiently differentiate between pre- and post-join publications, accounting for potential publication delays.

These individuals published 2,887 unique papers with 5,940 unique co-authors in the 4 years before joining the NI, and 4,147 unique papers with 9,160 unique co-authors in the 4 years after they joined the NI. Publication data, including details such as title, type, and journal/conference proceeding name, were collected using the Scopus publication database.

Researchers’ demographic information such as name, job position, year joined institution, and departmental affiliation, were provided through a roster of affiliates managed by the NI. We coded public-facing documents to identify the pronouns used to refer to each researcher, revealing a bias toward masculine pronouns in the sample (147 (70%) he/him, 45 (22%) she/her, and 16 (8%) non-gendered or missing public-facing gendered pronouns). Researchers held primary appointments in 53 unique academic departments at the University. Departmental membership was unevenly distributed such that a few departments contributed many NI members and many other departments contributed a few members. We cannot identify specific departments for privacy reasons, but the most conventional departments represented at the NI were biology departments (including animal, plant, and microbial foci), engineering, chemistry, and computer science disciplines. Less commonly represented departments included the social sciences and humanities. The majority of researchers in the dataset occupied tenure-track appointments in their home departments (71 (34%) Assistant Professor, 41 (20%) Associate Professor, 80 (38%) Full Professor, 16 (8%) other).

Data preprocessing was performed, including data cleaning, author and paper name disambiguation, and the creation of a paper-author network with associated attributes. Subsequent ego-centric network analysis and evaluation involved the extraction of key metrics of focal researchers authorship ego-networks for pre- and post- time periods such as unique paper and co-author counts, and various diversity indices based on departmental affiliations represented.

### Control group

We required a control group to assess whether joining the NI was the source of any observed effect, rather than other potentially exogenous factors (e.g. changes occuring naturally in researchers’ careers over time, selection effects that influenced researchers with a predisposition for diverse networks to join the NI, etc.). We also knew other important factors driving publication structures in our dataset would likely stem from the disciplines represented at the NI and researchers’ points in their professional career. As such, we sought a control group reproducing the distribution of departments and hiring dates within our NI sample as closely as possible, with the key exception that members of the control group would never join the NI during their careers. We adopted a matched-pairing strategy to select individuals from within the broader university context of the NI to meet these criteria. We first chose a subset of individuals (egos) from our primary dataset to replicate the distribution of departments found in our larger NI sample. From the NI sample, we examined the distribution of unique departments represented among the 208 NI researchers in the sample (n=53) to ensure that the sample for the control group followed a similar distribution. We aimed for a control group size of about 30% of our primary sample. Thus, we selected 60 individuals representing 34 departments to approximate the distribution of departments in the primary dataset. For departments at the tail end of the distribution, we randomly sampled to include some of these departments in the control group, but could not include control representation for each of the tail-departments while still approximating the department distribution of the primary dataset. For each ego in this subset, we identified researchers who joined the same department as the ego at a comparable time and who also possessed a 9-year record of publication in our database surrounding the ego’s start date at the NI (see section on Network Construction for justification of our choice of this 9-year window). Another requirement for inclusion as a control was that these researchers would not eventually join the NI. To offer a point of comparison to our primary dataset, we assigned each of these matched individuals a "start year" of the same year that their referential ego in the primary dataset joined the NI. We repeated the same network construction processes described in the “Network Construction” section above for the control group, and thus created 60x2 ego-networks 4 years pre-start date and 4 years post-start date. This approach allowed us to create matched pairs to investigate the impact of joining the NI on research outcomes while controlling for confounding variables such as time and institutional resources.

### Measures

Diversity indices are widely used in many scientific disciplines to quantify the distribution of types within a dataset and are perhaps most strongly associated with the field of ecology, where diversity indices are used to measure in a variety of ways the number and distribution of species in a community. Our previous work [35] reviewed diversity measures from communication, ecology, economics, and bibliometrics, and determined the three main metrics that were directly relevant to team science research: richness, evenness, and Shannon diversity index. In particular, we used the ecological operationalization of diversity as comprising two elements: the number of species in a community [i.e. richness [36] and their relative abundance [i.e. evenness [37]. Building on measures of richness and evenness, the Shannon diversity index [38] measures the evenness of the distribution of species within a system relative to its overall richness. This operationalization largely mirrors the concepts of variety, balance, and disparity discussed in prior works on interdisciplinary science [6,7]

We aimed to operationalize and measure three properties of interdisciplinarity, namely variety, balance, and disparity, [6–8], but due to substantial missing data on departmental affiliations for co-authors not affiliated with the NI, we could only reliably calculate variety. Therefore, in our primary analyses, we use richness (i.e. variety) as the main proxy for diversity of researchers’ collaboration networks, as it captures the breadth of unique connections, and had no missing data as it relies only on counts of papers and co-authors. This measurement approach complements and extends traditional bibliometric measures, which often focus on the variety of citation patterns [7,8,39], or number of disciplines presented [6,40]. While existing approaches measure interdisciplinarity through citation diversity or disciplinary variety at the paper or discipline level, we analyze diversity at the researcher level of analysis.

For descriptive purposes, the first section of our results describes the comparative departmental diversity in ego networks before and after joining NI. We do not compare the departmental diversity of the NI egos with the control group (described below) as departmental affiliation data were exclusively gathered for 394 NI-affiliated researchers in our complete dataset, so our calculation of departmental diversity between egos and alters was restricted to co-authorship collaborations among NI researchers. When interpreting these results, keep in mind that this analysis only examined co-authorship within the NI network (i.e. the 394 researchers in our primary dataset).

### Analysis

We conducted generalized linear mixed effects regression (GLMER) modeling with Poisson distribution to determine whether the changes in the richness of each researcher’s numbers of papers and co-authors were significantly different for NI affiliates versus the control group. Our modeling approach was selected after an extensive exploration of the dataset to assess appropriate analytic paradigms. Anonymized data and code used for this project are available at https://github.com/lydinh92/NI_Research. Additional methodological details about data processing and descriptive statistics for our sample are described in our S1 Appendix.

## Results

### Increase in researcher productivity and diversity of collaboration networks

We drew upon established measures of diversity from ecology (e.g. richness, Shannon evenness) to compare each researcher’s publication networks for the 4 years before and 4 years after joining the NI. Between their pre- and post-join networks, NI researchers had an average increase of 151% in the number of their publications (i.e. paper richness; from 15.6 to 23.7) and a 175% increase in the number of unique co-authors (i.e. co-author richness; from 35.5 to 62.1). After joining, Researchers co-authored with individuals in the NI network from more departments across the Institute’s campus (i.e. departmental richness; from 2.3 to 3.8 departments), and distributed their collaborations more equitably among those departments (i.e. median Shannon evenness increased from 0.791 to 0.895). These statistics suggest that researchers experienced substantial increases in the disciplinary diversity of their collaboration networks after joining the NI. However, because these descriptive results do not disentangle the effects of joining the NI from other potential exogenous factors, next we performed a comparison to a control group.

### Comparison to control group indicates NI as source of unique diversification effect

We leveraged the NI’s dual-membership appointment model to construct a control group that would allow us to isolate the effects of joining the NI versus other factors known to affect researcher productivity, namely institutional resources and time. We began by selecting 60 focal researchers from our primary dataset approximating the distribution of home departments represented at the NI. From university records, we selected an individual that joined the same home department, at a similar academic rank, at a similar time as each focal individual in the NI. Then, we assigned each control individual the same start date as when their focal individual joined the NI, to facilitate comparison of collaboration networks at similar time points in their careers. Thus, our control group consists of researchers with similar hiring dates, career phases, and departmental affiliations to our NI dataset with a primary difference being that control group researchers did not join the NI. Descriptive statistics for demographics of the control group are available in the S1 Appendix. Limitations in data availability only permitted access to measures of richness for the control group.

**Fig 1:**
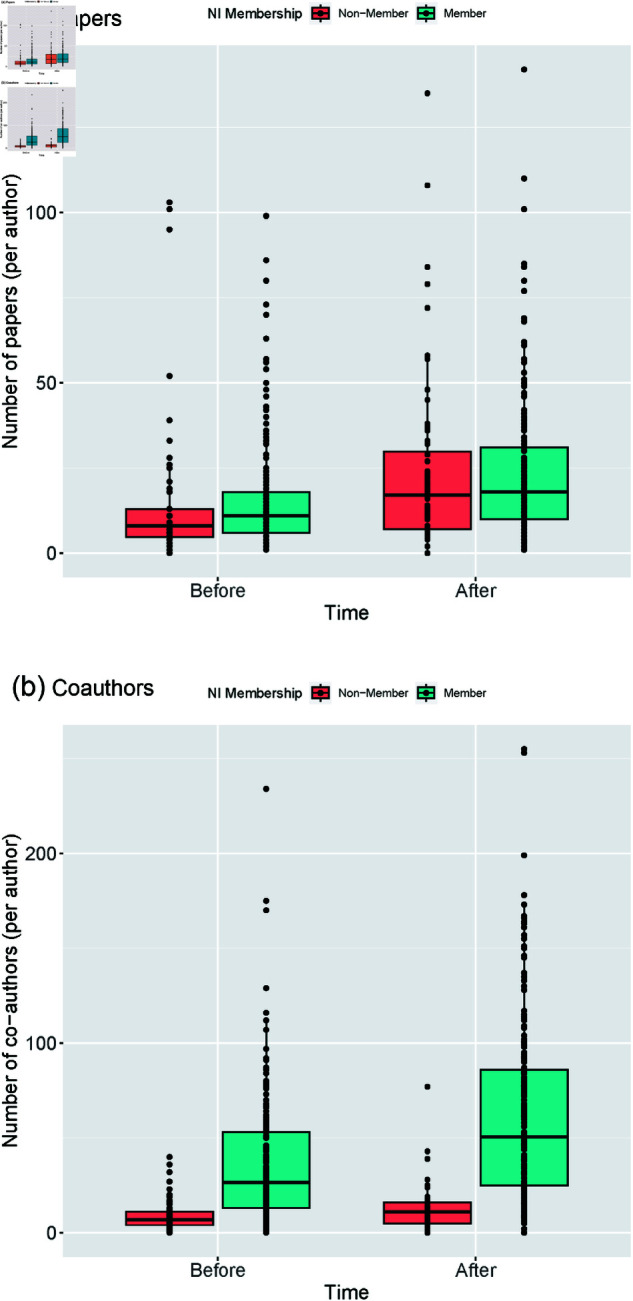
Differences in mean number of papers (top) and co-authors (bottom) depending on (1) NI membership and (2) time joined NI.

For both groups, paper richness and co-author richness increased over time (Fig 1). The control group is similar to the NI sample in terms of their initial research productivity (i.e. the number of papers they published in each time period) and its increase over time (Fig 1a). But, interesting differences emerge when comparing the makeup of each individual’s co-authorship networks (i.e. the number of unique co-authors with whom they published in each time period; Fig 1b).

**Fig 2:**
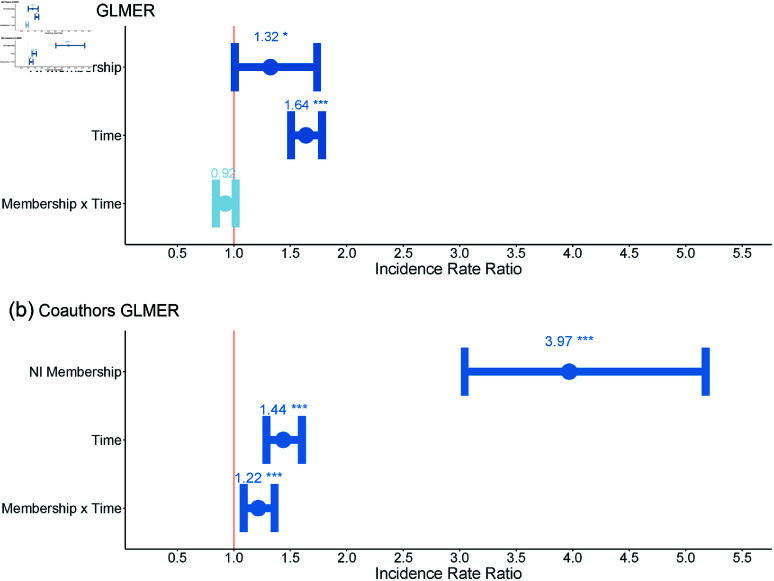
Regression estimates and incidence rate ratios from GLMER for number of papers (top) and coauthors (bottom).

Two generalized linear mixed effect (GLMER) models were used to compare the two groups in terms of paper richness (Model 1) and co-author richness (Model 2). Both models showed significant effects of NI membership (Model 1 IRR: 1.32, p=0.04; Model 2 IRR: 3.97, p<0.001) and Time (Model 1 IRR: 1.64, p<0.001; Model 2 IRR: 1.44, p<0.001) on paper and co-author richness (Fig 2). The second model also identified a significant interaction effect between Time and NI membership (IRR: 1.22, p=0.001; 95% CI: 1.09, 1.36), whereas the first model did not (IRR: 0.92, p=0.10; CI: 0.61, 1.33).

The significant model coefficients for NI membership indicate a selection effect wherein researchers who joined the NI exhibited a predisposition to higher productivity with a wider array of collaborators than members of the control group. The positive and significant incidence rates for NI membership in the GLMER predicts that an individual who would eventually join the NI tended to produce 32% more papers and publish with 297% more unique co-authors than an individual from the control group. Furthermore, the models both indicate significant effects for time, suggesting that regardless of NI membership, individuals publish more, and with more unique co-authors in the second time period.

The interaction effects across the two models, however, indicate that joining the NI produced an additional, unique influence on researchers above and beyond these effects. The absence of a significant interaction effect in Model 1 indicates that joining the NI did not result in any additional positive or negative influence on researchers’ paper productivity. The significant interaction coefficient in Model 2, however, indicates that researchers who joined the NI published with an increased diversity of co-authors after joining than would be expected by the other effects in the model. These effects suggest that the NI produced a significant influence on broadening researchers’ collaborations to include a wider variety of co-authors after joining the NI. These effects are particularly notable together: the models suggest that the increased diversity of NI researchers’ co-authorship networks was not simply driven by an increase in productivity, but through a disproportionate diversification of *who* they publish with after joining the NI.

One counter-explanation for this effect might arise from NI researchers simply publishing articles with substantially larger author lists after joining the NI. This was not the case. A secondary analysis indicated that although the size of co-author lists increased slightly over time across both NI and control groups, this effect was both small (an average increase of less than one co-author per paper for both NI and control groups) and the rate of change was commensurate for both NI and control groups.

## Discussion

Our case analysis of an interdisciplinary research institute offers quantitative evidence that access to resources dedicated to fostering interdisciplinarity can produce measurable positive effects on the diversity of researchers’ collaboration networks and no measurable negative effects on researchers’ productivity. The effects demonstrated here are particularly prescient given prior work indicating that interdisciplinary work is a laborious effort that can often hamper researchers’ productivity [10,17,21]. In particular, we show that interdisciplinary center membership can help researchers broaden their co-authorship network, and relatedly, increase their research outputs. The collaborative environment at the NI helps researchers maintain, and enhance, their productivity compared to peers working in traditional, discipline-specific setting. Compared to [21]’s study, which found that successful interdisciplinary research often takes additional time (up to five years of uninterrupted time) and effort to integrate diverse knowledge, we found that even within a four-year time span of joining the NI, researchers already saw measurable benefits for their scholarly productivity. This finding suggests that with institutional support and resources, like those at NI, researchers may reap the benefits of interdisciplinary research in less time than previously anticipated.

Researchers who joined the NI not only increased their co-authorship networks significantly more than those who did not, but also had significantly larger co-authorship networks before joining (Fig 1b). From these analyses we are not able to discern the mechanisms responsible for this pattern, but there are multiple possible explanations. Researchers who joined the NI may be predisposed to interdisciplinary collaboration, as indicated by their larger pre-join co-authorship networks. However, this does not preclude that the NI further expanded these networks by providing institutional resources that support interdisciplinary collaboration. The expansion of post-join co-author networks may be attributable to the NI forging connections among researchers from historically separate disciplines, or may be attributable to provisioning of key institutional resources, including access to advanced technologies or dedicated interdisciplinary workspaces. Thus, the findings raise important questions for future research regarding the mechanisms underlying the observed effects. When effective, do the resources provided by institutes like the NI serve to *generate* new scientific collaborations or, do they *amplify* individuals’ predetermined motivation, intent, or capacity to engage in collaborative work?

Understanding the mechanisms by which interdisciplinary research institutes promote new collaboration networks is a critical frontier in team science. Furthermore, researchers who joined the NI did not experience reduced productivity in the time period after joining the NI (Fig 1a), as posited could be the case by the interdisciplinary science literature. This lack of inhibition may also be attributed to the resources provided by the institute such as proactive administrative support, funding to support graduate researchers, and coordinated advice giving among networks of interdisciplinary labs. But, the single-site nature of our dataset also preclude us from identifying *which* mechanisms prevented this effect and *how* they did so. Another key area for future research will be to explore how institutional resources buffer interdisciplinary scientists from the inhibiting effects on research productivity documented elsewhere in the literature.

Although these findings are promising for the prospects of interdisciplinary research institutes, they are also limited by the single-site nature of our analysis. Here, we are not able to disentangle whether these effects are unique to the NI, or would apply to other scientific institutes dedicated to promoting interdisciplinary collaboration. For example, we cannot determine whether the resources provided by the NI would be as beneficial in disciplinary contexts whose inquiry is less reliant upon measurement technologies [41]. But, these results coincide with and extend recent findings that show the value of devoting explicit institutional resources to enabling interdisciplinary research [22–24]. Whereas prior work has emphasized the value of support resources at the project level of analysis, our findings suggest that resources can also produce enduring influences on researchers’ careers. As such, the findings presented here serve as further evidence that institutions that wish to enable interdisciplinary science should acknowledge that doing so requires dedicated and enduring resources to counter systemic and social pressures inhibiting interdisciplinarity.

We also recognize that interdisciplinarity is a multi-dimensional construct that includes variety, balance, and disparity [6]. As mentioned in the methods section, our study is limited to measuring variety in the analysis of interdisciplinarity due to substantial missing data for co-authors’ departmental affiliations. In future work, we aim to expand to our dataset by using Scopus API to obtain departmental affiliation data for co-authors outside of the NI. With these data, we will be able to compute measures of balance via evenness (i.e., the extent to which co-authors are evenly distributed across departments in each ego’s network) and disparity using Porter and Rafols’ [8] similarity matrix, which captures the cosine similarity between departmental pairs in our dataset.

Clearly, an important next step will involve applying these methods toward a multiple site comparative analysis. Such a cross-institutional comparative analysis would enable important secondary analysis to isolate how specific factors such as administrative structures and forms of resources affect researchers’ collaborations. Further, another important avenue for future research will include examining how the observed effects co-vary depending on secondary demographic variables that have been identified as consequential in interdisciplinary science, such as researcher’s home discipline, tenure, gender, and racial ancestry. The possibilities associated with dedicated interdisciplinary resources are strong, but we are only beginning to understand nuances of the dynamics by which institutions can successfully enable interdisciplinary science.

## Supporting information

S1 Appendix Contains detailed descriptions of data cleaning and preprocessing, sample descriptives, and GLMER Specification and Residual Diagnostics.
(PDF)

## References

[1] Wu L, Wang D, Evans JA. Large teams develop and small teams disrupt science and technology. Nature. 2019;566:379–82. doi: 10.1038/s41586-019-0941-9 30760923

[2] Zeng A, Fan Y, Di Z, Wang Y, Havlin S. Fresh teams are associated with original and multidisciplinary research. Nat Hum Behav. 2021;5(10):1314–22. doi: 10.1038/s41562-021-01084-x 33820976

[3] Shi F, Evans J. Surprising combinations of research contents and contexts are related to impact and emerge with scientific outsiders from distant disciplines. Nat Commun. 2023;14:1641. doi: 10.1038/s41467-023-36741-4 36964138 PMC10039062

[4] Uzzi B, Mukherjee S, Stringer M, Jones B. Atypical combinations and scientific impact. Science. 2013;342(6157):468–72. doi: 10.1126/science.1240474 24159044

[5] Venturini S, Sikdar S, Rinaldi F, Tudisco F, Fortunato S. Collaboration and topic switches in science. Sci Rep. 2024;14(1):1258. doi: 10.1038/s41598-024-51606-6 38218965 PMC10787828

[6] Stirling A. A general framework for analysing diversity in science, technology and society. J R Soc Interf. 2007;4(15):707–19. doi: 10.1098/rsif.2007.0213 17327202 PMC2373389

[7] Fontana M, Iori M, Montobbio F, Sinatra R. New and atypical combinations: an assessment of novelty and interdisciplinarity. Res Policy. 2020;49(7):104063. doi: 10.1016/j.respol.2020.104063

[8] Porter A, Rafols I. Is science becoming more interdisciplinary? Measuring and mapping six research fields over time. Scientometrics. 2009;81(3):719–45.

[9] Schilling MA, Green E. Recombinant search and breakthrough idea generation: an analysis of high impact papers in the social sciences. Res Policy. 2011;40:1321–31. doi: 10.1016/j.respol.2011.06.009

[10] Leahey E, Beckman CM, Stanko TL. Prominent but less productive. Admin Sci Q. 2017;62(1):105–39. doi: 10.1177/0001839216665364

[11] Barley WC, Dinh L, Workman H, Fang C. Exploring the relationship between interdisciplinary ties and linguistic familiarity using multilevel network analysis. Commun Res. 2022;49(1):33–60. doi: 10.1177/0093650220926001

[12] Fini R, Jourdan J, Perkmann M, Toschi L. A new take on the categorical imperative: gatekeeping, boundary maintenance, and evaluation penalties in science. Org Sci. 2023;34(3):1090–110. doi: 10.1287/orsc.2022.1610

[13] Bromham L, Dinnage R, Hua X. Interdisciplinary research has consistently lower funding success. Nature. 2016;534(7609):684–7. doi: 10.1038/nature18315 27357795

[14] Krauss A. Science’s greatest discoverers: a shift towards greater interdisciplinarity, top universities and older age. Humanit Soc Sci Commun. 2024;11(1):1–11. doi: 10.1057/s41599-024-02781-4

[15] Cummings JN, Kiesler S. Coordination costs and project outcomes in multi-university collaborations. Res Policy. 2007;36(10):1620–34. doi: 10.1016/j.respol.2007.09.001

[16] Andalón M, de Fontenay C, Ginther DK, Lim K. The rise of teamwork and career prospects in academic science. Nat Biotechnol. 2024;42(8):1314–9. doi: 10.1038/s41587-024-02351-8 39143163

[17] Marzano M, Carss DN, Bell S. Working to make interdisciplinarity work: Investing in communication and interpersonal relationships. J Agric Econ. 2006;57(2):185–97.

[18] National Academy of Sciences, Engineering, and Medicine. Facilitating interdisciplinary research. Washington, DC: The National Academies Press; 2005. Available from: https://nap.nationalacademies.org/catalog/11153/facilitating-interdisciplinary-research

[19] Hall KL, Stokols D, Stipelman BA, Vogel AL, Fend A, Masimore B, et al. Assessing the value of team science: a study comparing center- and investigator-initiated grants. Am J Prevent Med. 2012;42(2):157–63. doi: 10.1016/j.amepre.2011.10.011 PMC3586819 22261212

[20] Hackett EJ, Leahey E, Parker JN, Rafols I, Hampton SE, Corte U, et al. Do synthesis centers synthesize? A semantic analysis of topical diversity in research. Res Policy. 2021;50(1):104069. doi: 10.1016/j.respol.2020.104069 33390628 PMC7695893

[21] Laudel G, Gläser J. Beyond breakthrough research: epistemic properties of research and their consequences for research funding. Res Policy. 2014;43(7):1204–16.

[22] Stephens B, Downer JB, Cummings JN. Teamwork coaching in the research development process. Small Group Res. 2024; 55(6). doi: 10.1177/1046496424124072 PMC11684781 39737212

[23] Jones MS, Cravens AE, Zarestky J, Ngai C, Love HB. Facilitating psychological safety in science and research teams. Humanit Soc Sci Commun. 2024;11(1):1–12. doi: 10.1057/s41599-024-04037-7

[24] Cravens AE, Jones MS, Ngai C, Zarestky J, Love HB. Science facilitation: navigating the intersection of intellectual and interpersonal expertise in scientific collaboration. Humanit Soc Sci Commun. 2022;9(1):1–13. doi: 10.1057/s41599-022-01217-1

[25] Bowker GC, Timmermans S, Clarke AE, Balka E, editors. Boundary objects and beyond. MIT Press; 2016. Available from: https://mitpress.mit.edu/9780262528085/boundary-objects-and-beyond/

[26] Star SL, Ruhleder K. Steps towards an ecology of infrastructure: complex problems in design and access for large-scale collaborative systems. Proceedings of the 1994 ACM conference on computer supported cooperative work. 1994. p. 253–64.

[27] Nicolini D, Mengis J, Swan J. Understanding the role of objects in cross-disciplinary collaboration. Org Sci. 2012;23(3):612–29. doi: 10.1287/orsc.1110.0664

[28] Galison P. Image and logic: A material culture of microphysics. University of Chicago Press; 1997.

[29] Cetina KK. Epistemic cultures: How the sciences make knowledge. Harvard University Press; 1999.

[30] Biancani S, Dahlander L, McFarland DA, Smith S. Superstars in the making? The broad effects of interdisciplinary centers. Res Policy. 2018;47(3):543–57. doi: 10.1016/j.respol.2018.01.014

[31] Abramo G, Cicero T, D’Angelo CA. Assessing the varying level of impact measurement accuracy as a function of the citation window length. J Inform. 2011;5(4):659–67. doi: 10.1016/j.joi.2011.06.004

[32] Wang J. Citation time window choice for research impact evaluation. Scientometrics. 2013;94(3):851–72. doi: 10.1007/s11192-012-0775-9

[33] Björk BC, Solomon D. The publishing delay in scholarly peer-reviewed journals. J Inform. 2013;7(4):914–23. doi: 10.1016/j.joi.2013.09.001

[34] Tort AB, Targino ZH, Amaral OB. Rising publication delays inflate journal impact factors. PLoS One. 2012;7(12):e53374. doi: 10.1371/journal.pone.0053374 23300920 PMC3534064

[35] Dinh L, Barley WC, Johnson LP, Allan BF. Diversity measures for scientific collaborations. iConference 2023 proceedings. 2023.

[36] Whittaker RH. Evolution and measurement of species diversity. Taxon. 1972;21(2–3):213–51.

[37] Magurran AE. Ecological diversity and its measurement. Princeton University Press; 1988.

[38] Shannon CE. A mathematical theory of communication. Bell Syst Techn J. 1948;27(3):379–423. doi: 10.1002/j.1538-7305.1948.tb01338.x

[39] Yegros-Yegros A, Rafols I, D’Este P. Does interdisciplinary research lead to higher citation impact? The different effect of proximal and distal interdisciplinarity. PLoS One. 2015;10(8):e0135095. doi: 10.1371/journal.pone.0135095 26266805 PMC4534379

[40] Rafols I, Meyer M. Diversity and network coherence as indicators of interdisciplinarity: case studies in bionanoscience. Scientometrics. 2010;82(2):263–87. doi: 10.1007/s11192-009-0041-y

[41] Collins R. Why the social sciences won’t become high-consensus, rapid-discovery science. Sociol Forum. 1994;9(2):155–77. doi: 10.1007/BF01476360

