# Supplementary material for: Membership in team science institute enhances diversity of researchers’ collaboration networks
Source: PLoS One. 2025 May 23;20(5):e0322943. doi: 10.1371/journal.pone.0322943 (PMC12101652; doi:10.1371/journal.pone.0322943)
Supplement: S1 Appendix — Contains detailed descriptions of data cleaning and preprocessing, sample descriptives, and GLMER Specification and Residual Diagnostics. (PDF) [file pone.0322943.s001.pdf]

Membership in team science institute enhances diversity of researchers' collaboration networks

William C. Barley[1*], Ly Dinh[2], Lauren P. Johnson[1], Brian F. Allan[3]

1 Department of Communication, University of Illinois Urbana-Champaign, Urbana, Illinois, United States of America
2 School of Information Science, University of South Florida, Tampa, Florida, United States of America
3 Department of Entomology, University of Illinois Urbana-Champaign, Urbana, Illinois, United States of America

* Corresponding author
Email: barley@illinois.edu

Supporting information

S1 Appendix.

Data Cleaning & Preprocessing

The NI was identified as an ideal site to examine collaborative network dynamics for two reasons: (1) The NI provides extensive administrative support and plays an active role in forming interdisciplinary teams where affiliates from diverse disciplines come together to solve a common set of problems in the biological sciences, and (2) In their capacity as NI researchers, the Institute does not impose substantial additional responsibilities beyond a researcher's primary research. Thus, the primary objectives of NI researchers involve working on interdisciplinary collaborative projects. The researcher membership dataset was initially supplied to us by NI administration and contained information about 414 researchers. We determined 394 of these researchers had publications indexed in Scopus, and 208 had publications during the required 9-year window before and after joining the NI. The research team manually cleaned and verified these data with a number of rules that applied to each of 15 categories of demographic information represented therein. We utilized a publicly accessible institutional record of academic appointments to verify information for each NI researcher represented in the dataset, and corrected minor errors such as spelling mistakes in their full names. Information about a researcher's most recent degree (i.e. year of degree obtained and field of highest degree) were verified based on public-facing information stated on each researcher's faculty website, personal webpage/CV, or professional website (e.g. LinkedIn). Details about a researcher's job at the university and at NI (e.g. year joined institution, year joined NI, year left NI (if applicable), departmental affiliation) were validated using the institutional record of academic appointments, as well as faculty website(s). In particular, for researchers with multiple department affiliations, we included up to three department names, along with the associated full-time equivalent (FTE, e.g. 1 for 100% time, 0.5 for 50% time) stated in the institutional record. The bibliometric dataset was cleaned and preprocessed to ensure data were suitable for network analysis. Cleaning was conducted using Python Pandas package, and involved (1) converting encoding to UTF-8; (2) removing whitespaces; and (3) removing special characters. Next, we

disambiguated authors based on their email addresses and disambiguated papers based on multiple characteristics. To disambiguate papers, we created a set of guidelines to identify a paper as unique. Each paper in our database included the following information: year published, full title, publication type (e.g., journal, book, conference proceeding), journal name, DOI, and author list. Papers that shared at least five of the six characteristics were coded as the same paper, while papers that differed in more than one category were coded as unique. Our disambiguation process yielded a total of 7,034 unique papers, where 2,887 papers were published by the 208 authors in the 4-years before joining the NI, and 4,147 papers in the 4-years after joining the NI. Disambiguation was a crucial preprocessing type for our dataset as we had instances of authors who had the exact same name, but different email addresses (e.g. John Doe (jdoe@ni.edu) & John Doe (j.doe@midu.edu)). With the resulting paper-author adjacency list, we then excluded 'Patents' from the list of permitted publication types, leaving 27 unique types of publications in the dataset (e.g. Book/Report/Conference proceeding – Book; Chapter in Book/Report/Conference proceeding - Conference contribution; Contribution to journal - Special issue, etc.). Among the 27 publication types, 79% of the papers were peer-reviewed journal articles, 9% of the papers were peer-reviewed conference proceedings, and 5% of the papers were review articles.

We also excluded papers with more than 30 authors from the dataset to address the potential influence of hyperauthored papers, using the threshold method we developed in [1]. Our prior study found that the inclusion of a relatively small number of hyperauthored papers very disproportionately influences the structural positioning of central authors and the topological characteristics of the co-authorship network. This approach consists of three steps: (1) checking the normality of the authors per paper distribution, (2) detecting outliers using the empirical rule for normal distributions or Chebyshev's rule for skewed distributions, and (3) cross-validating the cutoff point with the cumulative frequency distribution approach. This process excluded 256 hyperauthored papers (with number of authors ranging from 31 to 317).

Lastly, we converted the paper-author adjacency list to a paper-author edgelist where an edge represented a paper that belonged to an author. We assigned each paper and author a unique paper_ID and author_ID so that the attributes of each paper and author (obtained from the researcher membership dataset) were linked to the correct paper-author edge.

Sample Descriptives

Table S1 shows the descriptive statistics for NI and Non-NI researchers, including departmental affiliations, job titles, gender distribution, and NI join dates. The two groups are mostly similar in key characteristics, such as join dates and gender distribution.

GLMER Specification and Residual Diagnostics

As described in the main text, two generalized linear mixed-effects regression (GLMER) models were calculated to estimate the influence of NI membership, time, and interaction effects on a researchers numbers of unique papers (i.e. paper richness; model 1) and on researchers counts of unique co-authors (i.e. co-author richness; model 2). Parameter estimates for these models are reported in figure 2 of the main document. In the first model for the number of unique papers, NI Membership has a significant positive effect (0.28, SE = 0.14, 95% CI: 0.01 - 0.55; p=0.04), suggesting that NI Membership is associated with a greater number of papers compared to the control group. Time shows a highly significant positive effect (0.49, SE = 0.04, 95% CI: 1.51 - 1.78; p=0.000), indicating that for both groups, the number of papers rises over time.

Group	Unique Depts	Job Titles (%)	Gender	NI Join Date
NI	52	Assistant Professor: 34.13% (71)<br>Associate Professor: 19.71% (41)<br>Professor: 38.46% (80)<br>Others: 7.69% (16)	M: 59.62% (124)<br>F: 13.94% (29)<br>N/A: 26.44% (55)	Average: 2009<br>Range: 1998-2017
Non-NI	34	Assistant Professor: 66.67% (40)<br>Associate Professor: 16.67% (10)<br>Professor: 16.67% (10)	M: 63.33% (38)<br>F: 21.67% (13)<br>N/A: 15.0% (9)	Average: 2009<br>Range: 2004-2017

Table S1. Descriptive statistics for departments, job titles, gender, and join dates for NI and Non-NI researchers

However, the interaction between NI Membership and Time is non-significant (-0.08, SE = 0.05, 95% CI: -0.18 - 0.02; p=0.10), with a negative estimate signifying a limited impact of the interaction effect of NI Membership and Time on the number of papers. In the second model for the number of unique co-authors, NI Membership has a highly significant positive effect (1.38, SE = 0.14, 95% CI: 1.11 - 1.65; p=0.000), indicating that being a member at NI is associated with a greater number of co-authors compared to researchers in the control group who are not members of NI. Time exhibits a highly significant positive effect (0.36, SE = 0.06, 95% CI: 0.25 - 0.47; p = 0.000), suggesting that for both groups, the expected number of co-authors increases over time. Furthermore, the interaction effect between NI Membership and Time has a significant positive effect (0.20, SE = 0.06, 95% CI: 0.08 - 0.31; p = 0.001), highlighting the number of co-authors increases significantly more for NI Members after joining NI.

For each of the two models, we first confirmed that a Poisson distribution for generalized linear mixed-effects model was suitable using both the histogram and quantile-quantile (QQ) plot. We found that the distributions were not non-normally distributed. Therefore, a generalized linear mixed-effects model was preferable over a linear mixed-effects model. Next, we utilized the diagnostics tools available in *R*'s *lme4* package to (1) plot the residual versus fitted values for each model output, and (2) compute model evaluation metrics, including R^2. First, the residual versus fitted values plot allowed us to examine the relationship between the model's predicted values and the model's errors. Residuals were randomly scattered around zero, suggesting that the model provided a reasonable fit to the data. The absence of a distinct pattern in the residuals also indicated that the linearity assumption between our independent variables and the link-transformed response variable was not violated. Second, we assessed model fit statistics for both models. In model 1, we found that the marginal R^2 was low (R^2=0.06), indicating that 6% of the variance in the number of papers was explained by our two fixed effects (NI membership and Time), and an interaction effect (NI membership x Time) was relatively small. On the other hand, a high conditional R^2 (0.92) suggested that the inclusion of random effects (the researcher), along with the fixed effects and an interaction term, explained 92% of the variance in the response variable. In other words, the variance was largely explained by the differences in numbers of papers across different researchers. We found similar patterns in model 2, with the exception that the marginal R^2 was higher than the previous model (R^2=0.37). This means that the two fixed effects along with an interaction term explained 37% of the variance in the number of co-authors. In other words, a substantial proportion of

the variance was explained by the effects of NI membership, time, and their interaction.

References

1. Dinh L, Barley WC, Johnson L, Allan BF. Hyperauthored papers disproportionately amplify important egocentric network metrics. Quantitative Science Studies. 2024; p. 1–24.
